# The efficacy of finerenone on hierarchical composite endpoint analysed using win statistics in patients with heart failure and mildly reduced or preserved ejection fraction: A prespecified analysis of FINEARTS‐HF

**DOI:** 10.1002/ejhf.3669

**Published:** 2025-04-29

**Authors:** Toru Kondo, Pardeep S. Jhund, Alasdair D. Henderson, Brian L. Claggett, Akshay S. Desai, Meike Brinker, James Lay‐Flurrie, Patrick Schloemer, Prabhakar Viswanathan, Flaviana Amarante, Chern‐En Chiang, Gerasimos Filippatos, Carolyn S.P. Lam, Mark C. Petrie, Michele Senni, Morten Schou, Subodh Verma, Adriaan A. Voors, Dirk von Lewinski, Faiez Zannad, Bertram Pitt, Muthiah Vaduganathan, Scott D. Solomon, John J.V McMurray

**Affiliations:** ^1^ British Heart Foundation Cardiovascular Research Centre University of Glasgow Glasgow UK; ^2^ Department of Cardiology Nagoya University Graduate School of Medicine Nagoya Japan; ^3^ Cardiovascular Division Brigham and Women's Hospital, Harvard Medical School Boston MA USA; ^4^ Cardiology and Nephrology Clinical Development, Bayer AG Wuppertal Germany; ^5^ Bayer plc, Research & Development, Pharmaceuticals Reading UK; ^6^ Bayer, Research & Development, Pharmaceuticals Berlin Germany; ^7^ Bayer, Research & Development, Pharmaceuticals Whippany NJ USA; ^8^ Bayer, Research & Development, Pharmaceuticals São Paulo Brazil; ^9^ General Clinical Research Center and Division of Cardiology Taipei Veterans General Hospital, and National Yang Ming Chiao Tung University Taipei Taiwan; ^10^ Department of Cardiology Attikon University Hospital, School of Medicine, National and Kapodistrian University of Athens Athens Greece; ^11^ National Heart Centre Singapore & Duke‐National University of Singapore Singapore City Singapore; ^12^ University of Milano‐Bicocca Milan Italy; ^13^ Papa Giovanni XXIII Hospital Bergamo Italy; ^14^ Department of Cardiology Herlev‐Gentofte University Hospital Hellerup Denmark; ^15^ St Michael's Hospital and University of Toronto Toronto ON Canada; ^16^ University Medical Center Groningen Groningen The Netherlands; ^17^ Division of Cardiology, Department of Internal Medicine Medical University of Graz Graz Austria; ^18^ Université de Lorraine, Inserm Clinical Investigation Centre, CHU Nancy France; ^19^ University of Michigan, School of Medicine Ann Arbor MI USA

**Keywords:** Heart failure, Finerenone, Clinical trial, Hierarchical composite endpoint, Win ratio, Win statistics

## Abstract

**Aims:**

FINEARTS‐HF demonstrated the efficacy of finerenone in reducing total worsening heart failure (HF) events (first and recurrent) and cardiovascular death, compared to placebo, in patients with HF and mildly reduced or preserved ejection fraction. We examined the effect of finerenone on these events according to their clinical importance using win statistics.

**Methods and results:**

We developed a prespecified hierarchical composite endpoint including the components of the original primary outcome: cardiovascular death (tier 1), total HF hospitalizations (tier 2), and total urgent HF visits (tier 3). For tiers 2 and 3, the number of events was analysed first, followed by the time‐to‐first event. Because win statistics are affected by the censoring distribution, we assessed the hierarchical composite outcome over a fixed period of 24 months. The 6001 participants analysed were randomized equally to finerenone (*n* = 3003) or placebo (*n* = 2998). At 24 months, a total of 825 cardiovascular deaths and worsening HF events were observed in the finerenone group, compared with 1012 events in the placebo group. The win ratio was 1.17 (95% confidence interval [CI] 1.04–1.32) (*p* = 0.010), demonstrating more wins than losses in the finerenone group. The win odds, corresponding to the treatment effect, was 1.05 (95% CI 1.01–1.09), and the net benefit, corresponding to the absolute risk difference, was 2.6% (95% CI 0.6–4.5%). The win ratio remained above 1.0 from 60 days after randomization and reached a plateau after approximately 12 months. HF hospitalizations contributed more to the overall results than cardiovascular death. The win odds at 12 months was 1.04 (95% CI 1.01–1.08), and when adding the Kansas City Cardiomyopathy Questionnaire total symptom score to the hierarchical endpoint as a continuous variable, that increased to 1.07, which is almost identical to the win ratio due to the decrease in ties.

**Conclusion:**

Finerenone treatment led to a significant improvement in a composite hierarchical outcome that incorporated cardiovascular death, total HF hospitalizations, and total urgent HF visits, with early onset of benefit.

Clinical Trial Registration: ClinicalTrials.gov ID NCT04435626.

## Introduction

Steroidal mineralocorticoid receptor antagonists (MRAs), including spironolactone and eplerenone, are beneficial in patients with heart failure (HF) and reduced ejection fraction and are strongly recommended in practice guidelines.^1^, ^2^, ^3^, ^4^ However, in patients with HF and mildly reduced or preserved ejection fraction (HFmrEF/HFpEF), spironolactone did not reduce the primary endpoint significantly, compared to placebo, in the TOPCAT trial (Treatment of Preserved Cardiac Function Heart Failure with an Aldosterone Antagonist).^5^ Recently, however, the new non‐steroidal MRA finerenone was shown to be superior to placebo in patients with HFmrEF/HFpEF enrolled in FINEARTS‐HF (Finerenone Trial to Investigate Efficacy and Safety Superior to Placebo in Patients with Heart Failure), with a significant 16% (95% confidence interval [CI] 5–26%) reduction in the primary composite outcome (*p* = 0.007).^6^ This endpoint consisted of total worsening HF events (HF hospitalizations and urgent HF visits) and cardiovascular deaths and was analysed using a semiparametric proportional rates method.^6^ This approach has the advantage of accounting for all fatal and non‐fatal events occurring during the follow‐up period, while the conventional time‐to‐first event analysis using a Cox proportional hazard model ignores many non‐fatal and fatal events occurring after the first non‐fatal event.^7^, ^8^, ^9^ However, even a total event approach has the limitation of giving equal statistical weight to clinically less important events (e.g. urgent HF visits) and more important events (e.g. cardiovascular deaths). Additionally, the measurement of health status (including symptom burden, physical limitations, and health‐related quality of life) at fixed points during the follow‐up period is challenging to incorporate into a composite morbidity/mortality outcome, using either method and is usually analysed as a separate secondary outcome. Win statistics, proposed by Pocock and colleagues, enable the evaluation of outcomes included in a hierarchical composite based on their clinical priority, and other continuous or categorical components such as patient‐reported outcomes and biomarkers can also be incorporated into such a composite.^10^ These advantages have led to increasing exploration of win statistics in recent clinical trials and even their use to analyse the primary endpoint.^8^, ^11^, ^12^, ^13^, ^14^, ^15^, ^16^, ^17^, ^18^, ^19^, ^20^, ^21^, ^22^, ^23^, ^24^, ^25^, ^26^, ^27^, ^28^

In this prespecified analysis, we used win statistics to explore the efficacy of finerenone in FINEARTS‐HF. We examined a prespecified hierarchical composite endpoint that considered the clinical importance of the events in the primary endpoint (ranked in order of clinical importance: cardiovascular death, HF hospitalization, and urgent HF visits), as well as the number of events and time to events. Furthermore, we evaluated the effect of finerenone using an extended hierarchical composite endpoint created by adding a patient‐reported outcome.

## Methods

FINEARTS‐HF was a prospective, double‐blind, randomized controlled trial in patients with HFmrEF/HFpEF, comparing the efficacy and safety of finerenone with a matching placebo, added to usual care. The trial designs and primary results are published.^6^, ^29^, ^30^ The trial protocol was approved by ethics committees at each investigative site and written informed consent was obtained from each patient.

### Study patients

Key inclusion criteria were age ≥40 years, New York Heart Association (NYHA) functional class II–IV symptoms, diuretic treatment for at least 30 days prior to randomization, left ventricular ejection fraction (LVEF) ≥40%, evidence of structural heart disease (left atrial enlargement or left ventricular hypertrophy), and elevated N‐terminal pro‐B‐type natriuretic peptide (or B‐type natriuretic peptide). The key exclusion criteria included an estimated glomerular filtration rate (eGFR) <25 ml/min/1.73 m^2^ and serum potassium level >5.0 mmol/L. Patients could be enrolled either as outpatients or in the setting of hospitalization for worsening HF.

Eligible patients were randomized in a 1:1 ratio to receive either finerenone or a matching placebo. If patients had an eGFR ≤60 ml/min/1.73 m^2^, treatment was started at a dose of 10 mg once daily with a maximum target maintenance dose of 20 mg once daily. If patients had an eGFR >60 ml/min/1.73 m^2^, treatment was started at a dose of 20 mg once daily with a maximum target maintenance dose of 40 mg once daily. Patients were screened between 14 September 2020 and 10 January 2023, and were followed until 14 June 2024.

### Study outcomes

The primary outcome for FINEARTS‐HF was a composite of total worsening HF events (first and recurrent) and cardiovascular death. Worsening HF events included either HF hospitalization or urgent HF visit. All deaths and possible worsening HF events were adjudicated by an independent clinical events committee.

In this prespecified analysis, we used a hierarchical composite endpoint including the components of the original primary outcome of FINEARTS‐HF, i.e. cardiovascular death (tier 1), total HF hospitalizations (tier 2), and total urgent HF visits (tier 3). For tiers 2 and 3, the number of events was analysed first, followed by an analysis of the time‐to‐first event. These events were compared over a fixed follow‐up period; censoring earlier than the defined fixed follow‐up period was considered censoring at the fixed follow‐up period to address the effect of censoring distributions on win statistics results.^10^, ^31^, ^32^, ^33^, ^34^ Fixed follow‐up periods for the main model were defined as 24 months (= 720 days in this study), based on an assessment of the censoring distribution in patients who did not die during follow‐up periods. Non‐cardiovascular death was treated as censoring. As a secondary analysis, we added the evaluation of the models with and without the Kansas City Cardiomyopathy Questionnaire total symptom score (KCCQ‐TSS), which is a patient‐reported outcome specific to HF and was a prespecified secondary outcome in FINEARTS‐HF. Scores range from 0 to 100, with a higher score indicating fewer symptoms and physical limitations. To allow the comparison between hierarchies with and without KCCQ‐TSS, we constructed four models which were evaluated at 12 months (= 360 days in this study) to utilize the KCCQ‐TSS at 12 months. For the 12‐month model without KCCQ‐TSS, we evaluated the same hierarchical composite endpoint including the same tiers used in the main model (second model). Further models incorporated KCCQ change from baseline to 12 months as an additional tier(s) analysed as a dichotomous variable (third model); as a dichotomous variable and a continuous variable (fourth model with a 4th and 5th tier); and as a continuous variable (fifth model). Finally, we evaluated a model (sixth model) including the full follow‐up for all patients, including the same hierarchical composite endpoint and the same tiers used in the main model. The models evaluated are summarized in online supplementary *Table* *S1*.

### Statistical analyses

Baseline characteristics were summarized by randomized groups as frequencies with percentages for categorical variables, means with standard deviation, or medians with interquartile ranges for continuous variables.

The unmatched win statistics method was used, in which every patient in the finerenone group was paired and compared with every patient in the placebo group^10^; pairs representing the product of the number of individuals in the finerenone group and placebo group were created and compared. Comparisons were made in descending order of tiers; once a tier was settled, the next tier was not evaluated, and if the last tier was not settled, the comparison pair was considered a tie.

In tier 1, the time to cardiovascular death was compared during a fixed follow‐up period. For the analysis of total events in tier 2–3, first, the number of events was compared, and then, the time‐to‐first event was compared during the fixed follow‐up period, i.e. 24 months for the main model and 12 months for the second to fifth models. For the third model including KCCQ‐TSS change, in tier 4, the change of KCCQ‐TSS from baseline to 12 months was evaluated as a dichotomous variable; pairs were compared on whether patients had ≥10‐point improvement, followed by ≥5‐point deterioration. Different thresholds for the KCCQ‐TSS change were used to define improvement (10 points) and deterioration (5 points), based on a recent anchor‐based analysis.^35^, ^36^ For the fourth model, the change of KCCQ‐TSS from baseline to 12 months as a continuous variable was added to the third model as the last tier (tier 5). Finally, for the fifth model, the change of KCCQ‐TSS as a continuous variable was added to the second model as the last tier (tier 4).

From the aforementioned comparisons of the pairs, the percentages of wins (*P*_W_), losses (*P*_L_), and tied pairs (*P*_T_) were obtained. We calculated three win statistics; win ratio, P_W_/P_L_; win odds, (*P*_W_ + 0.5*P*_T_)/(*P*_L_ + 0.5*P*_T_); and net benefit, *P*_W_−*P*_L_. The number needed to treat (NNT) was also obtained by the inverse of net benefit.^37^ Win ratio and win odds >1 demonstrate that there are more ‘wins’ with finerenone compared to placebo. The method of Pocock and colleagues and the corresponding variances based on the U‐statistic method of Dong *et al*. were used to compute the win ratio.^10^, ^38^ The win ratio with 95% CI, the win odds with 95% CI, and the percentages of wins or losses over time were plotted for every 10‐day fixed follow‐up period for the hierarchical model based on the main model (i.e. tier 1, cardiovascular death; tier 2, total HF hospitalizations; tier 3, total urgent HF visits).^39^ Because KCCQ‐TSS represents the outcome at 12 months only, making it difficult to assess win ratios over time in tier 4, the plots were drawn only for tiers 1–3. Stratified win statistics were calculated based on the strata used in randomization, i.e. geographic region and baseline LVEF (<60% or ≥60%). The win statistics of the main model according to prespecified subgroups were also computed.

Multiple imputation was applied for missing KCCQ‐TSS values at 12 months because assuming missing values are ties can bias the win statistics to the null. We performed reference‐based imputation using observed data to make reliable assumptions about the joint distribution of observed and missing values over time.^40^, ^41^ We used the ‘copy increments in reference’ method so assumed a joint distribution of observed and missing data is multivariate normal with a mean vector from the observed data (up to the last observation) and mean increments for unobserved (missing) values that follow those from the placebo group. We derived 10 imputed datasets, with missing values imputed based on observed KCCQ‐TSS values, treatment group assignment, study visit of missing value, and baseline values for age, sex, race, region, body mass index, systolic blood pressure, eGFR, atrial fibrillation at baseline electrocardiography, previous or ongoing HF hospitalization, NYHA functional class, LVEF, history of hypertension, history of myocardial infarction, history of diabetes, and history of stroke. Imputation was performed using the mimix package in STATA.^40^ Patients without baseline KCCQ‐TSS at baseline were excluded from the analyses of the third, fourth, and fifth models (*n* = 15).

To compare with the win statistics analyses, we conducted the conventional statistical approaches to compute rate ratios (RR) for total (first and recurrent) events using the Lin, Wei, Yang and Ying (LWYY) method and hazard ratios (HR) for time‐to‐first event analyses using Cox proportional hazard models with follow‐up truncated at 12 and 24 months, both of which were stratified by geographic region and baseline LVEF (<60% or ≥60%). The difference in the change in KCCQ‐TSS from baseline to 12 months between treatment groups was analysed using mixed‐effect models for repeated measurements adjusted for baseline KCCQ‐TSS, visit (month 6, 9, and 12), treatment assignment, geographic region, baseline LVEF (<60% or ≥60%), and interaction of treatment and visit. The difference in proportions of KCCQ‐TSS 10‐point improvement or 5‐point deterioration between treatment groups was compared by logistic regression model adjusted for geographical region, baseline LVEF (<60% or ≥60%), and baseline KCCQ‐TSS.

All analyses were conducted using STATA version 18.0 (Stata Corp., College Station, TX, USA) and the WINS package in R version 4.4.0.^42^

## Results

### Patients

The 6001 participants analysed were randomized equally to finerenone (*n* = 3003) and placebo (*n* = 2998) (*Table* *1*). The median follow‐up was 32 months (interquartile range, 23 to 37). At our prespecified fixed follow‐up time of 24 months, a total of 825 cardiovascular deaths and worsening HF events were observed in the finerenone group, compared with 1012 events in the placebo group; and, at 12 months, 456 events were observed in the finerenone group and 588 in the placebo group (*Table* *2*).

**Table 1:** Characteristics of the patients at baseline

	Finerenone (*n* = 3003)	Placebo (*n* = 2998)
Age, years	71.9 ± 9.6	72.0 ± 9.7
Female sex, *n* (%)	1355 (45.1)	1377 (45.9)
Region, *n* (%)		
Western Europe, Oceania and others	624 (20.8)	632 (21.1)
Eastern Europe	1329 (44.3)	1321 (44.1)
Asia	493 (16.4)	490 (16.3)
North America	235 (7.8)	236 (7.9)
Latin America	322 (10.7)	319 (10.6)
Race, *n* (%)		
White	2366 (78.8)	2369 (79.0)
Black	49 (1.6)	39 (1.3)
Asian	497 (16.6)	499 (16.6)
Other	91 (3.0)	91 (3.0)
Body mass index, kg/m^2^	29.9 ± 6.1	30.0 ± 6.1
Body mass index category, *n* (%)		
<18.5 kg/m^2^	33 (1.1)	32 (1.1)
18.5–<25 kg/m^2^	629 (21.0)	612 (20.5)
25–<30 kg/m^2^	996 (33.2)	994 (33.2)
30–<35 kg/m^2^	767 (25.6)	779 (26.0)
≥35 kg/m^2^	571 (19.1)	575 (19.2)
Vital signs		
Heart rate, bpm	71.7 ± 11.8	71.2 ± 11.9
In patients without AF at baseline ECG	68.2 ± 10.1	68.5 ± 10.4
In patients with AF at baseline ECG	77.1 ± 12.2	75.9 ± 12.6
Systolic blood pressure, mmHg	129.5 ± 15.3	129.3 ± 15.3
Diastolic blood pressure, mmHg	75.6 ± 10.3	75.4 ± 10.4
HF characteristics		
Previous or ongoing HF hospitalization, *n* (%)	1797 (59.8)	1822 (60.8)
Time since index HF event, *n* (%)		
Randomized during/at HF event	389 (13.0)	360 (12.0)
≤7 days from randomization	220 (7.3)	250 (8.3)
>7 days–≤3 months	1030 (34.3)	998 (33.3)
>3 months	448 (14.9)	489 (16.3)
No index HF event	916 (30.5)	901 (30.1)
NYHA functional class, *n* (%)		
II	2081 (69.3)	2065 (68.9)
III	903 (30.1)	910 (30.4)
IV	18 (0.6)	23 (0.8)
KCCQ‐TSS, points^a^	67.6 ± 23.9	66.5 ± 23.9
History of LVEF <40%, *n* (%)	147 (4.9)	126 (4.2)
LVEF, %	52.6 ± 7.8	52.5 ± 7.8
LVEF category, *n* (%)		
<50%	1093 (36.5)	1079 (36.0)
≥50%–<60%	1329 (44.3)	1345 (44.9)
≥60%	575 (19.2)	572 (19.1)
AF at baseline ECG, *n* (%)	1165 (38.8)	1128 (37.6)
Laboratory values		
HbA1c, %	6.4 ± 1.2	6.4 ± 1.2
Creatinine, mg/dl	1.1 ± 0.3	1.1 ± 0.4
eGFR, ml/min/1.73 m^2^	61.9 ± 19.4	62.3 ± 20.0
eGFR category, *n* (%)		
<60 ml/min/1.73 m^2^	1451 (48.3)	1437 (47.9)
≥60 ml/min/1.73 m^2^	1552 (51.7)	1561 (52.1)
Potassium, mmol/L	4.4 ± 0.5	4.4 ± 0.5
Urine albumin to creatinine ratio, mg/g	18 (7–67)	19 (7–66)
NT‐proBNP, pg/ml	1052 (467–1937)	1028 (433–1963)
In patients without AF at baseline ECG	596 (321–1239)	582 (307–1299)
In patients with AF at baseline ECG	1724 (1157–2772)	1712 (1141–2814)
Clinical history, *n* (%)		
AF or atrial flutter	1669 (55.6)	1650 (55.0)
Diabetes	1217 (40.5)	1222 (40.8)
Coronary artery disease	1143 (38.1)	1157 (38.6)
Myocardial infarction	784 (26.1)	757 (25.3)
Stroke	427 (14.2)	404 (13.5)
Coronary artery bypass graft	460 (15.3)	456 (15.2)
Percutaneous coronary intervention	746 (24.8)	725 (24.2)
Hypertension	2640 (87.9)	2685 (89.6)
Chronic obstructive pulmonary disease	380 (12.7)	393 (13.1)
Medical therapy, *n* (%)		
ACEi	1083 (36.1)	1072 (35.8)
ARB^b^	1054 (35.2)	1047 (34.9)
ARNI	256 (8.5)	257 (8.6)
ACEi, ARB, or ARNI	2379 (79.2)	2380 (79.4)
Beta‐blocker	2541 (84.6)	2554 (85.2)
SGLT2i	393 (13.1)	424 (14.1)
Loop diuretic	2618 (87.2)	2621 (87.4)

Data are presented as mean ± standard deviation or median (interquartile range) for continuous measures, and *n* (%) for categorical measures.

ACEi, angiotensin‐converting enzyme inhibitor; AF, atrial fibrillation; ARB, angiotensin receptor blocker; ARNI, angiotensin receptor–neprilysin inhibitor; ECG, electrocardiogram; eGFR, estimated glomerular filtration rate; HbA1c, glycated haemoglobin; KCCQ‐TSS, Kansas City Cardiomyopathy Questionnaire total symptom score (range from 0 to 100, with a higher score indicating fewer symptoms and physical limitations); LVEF, left ventricular ejection fraction; NT‐proBNP, N‐terminal pro‐B‐type natriuretic peptide; NYHA, New York Heart Association; SGLT2i, sodium–glucose co‐transporter‐2 inhibitor.

^a^ Obtained from non‐imputation dataset.

^b^ Excluding ARNI.

**Table 2:** Win ratio analyses: outcomes included in the hierarchical composite outcome

	Number of events		Rate per 100 patient‐years (95% CI)		RR, HR, difference in means, OR (95% CI)
	Finerenone (*n* = 3003)	Placebo (*n* = 2998)	Finerenone (*n* = 3003)	Placebo (*n* = 2998)	
At 24 months					
Total worsening HF events (first and recurrent) and cardiovascular death^a^	825	1012	11.2 (10.1–12.4)	13.8 (12.5–15.2)	0.81 (0.71–0.93)
Cardiovascular death^b^	174	192	3.2 (2.7–3.7)	3.5 (3.1–4.1)	0.91 (0.74–1.11)
Total HF hospitalizations (first and recurrent)^a^	575	703	7.8 (6.9–8.8)	9.6 (8.5–10.7)	0.81 (0.69–0.95)
Total urgent HF visits (first and recurrent)^a^	76	118	1.0 (0.8–1.3)	1.6 (1.3–2.0)	0.64 (0.45–0.90)
At 12 months					
Total worsening HF events (first and recurrent) and cardiovascular death^a^	456	588	6.2 (5.4–7.0)	8.0 (7.1–9.0)	0.77 (0.66–0.91)
Cardiovascular death^b^	97	97	3.3 (2.7–4.1)	3.3 (2.7–4.1)	1.00 (0.76–1.33)
Total HF hospitalizations (first and recurrent)^a^	315	409	4.3 (3.7–4.9)	5.6 (4.9–6.4)	0.77 (0.63–0.93)
Total urgent HF visits (first and recurrent)^a^	44	83	0.6 (0.4–0.8)	1.1 (0.9–1.4)	0.53 (0.35–0.80)
KCCQ‐TSS change from baseline to 12 months, points^c^, ^d^	8.0 ± 0.3	6.4 ± 0.3	–	–	1.6 (0.8–2.4)
KCCQ‐TSS improvement ≥10 points, *n* (%)^d^, ^e^	1398/2846 (49.1%)	1354/2834 (47.8%)			1.08 (0.97–1.20)
KCCQ‐TSS deterioration ≥5 points, *n* (%)^d^, ^e^	764/2846 (26.8%)	801/2834 (28.3%)			0.92 (0.81–1.03)

All statistics were stratified by geographical regions and left ventricular ejection fraction (<60% or ≥60%). 1 year = 360 days.

CI, confidence interval; HF, heart failure; HR, hazard ratio; KCCQ‐TSS, Kansas City Cardiomyopathy Questionnaire total symptom score; OR, odds ratio; RR, rate ratio.

^a^ RR are shown.

^b^ HR are shown.

^c^ Difference in means is shown.

^d^ Results from the main analysis dataset shown without further imputation for missingness due to loss to follow‐up.

^e^ OR are shown.

The KCCQ‐TSS increased (improved) by 8.0 ± 0.3 points in the finerenone group and by 6.4 ± 0.3 points in the placebo group from baseline to 12 months (difference, 1.6 [95% CI 0.8–2.4] points).

### Results of win statistics for the hierarchical composite endpoints

**Figure 1:**
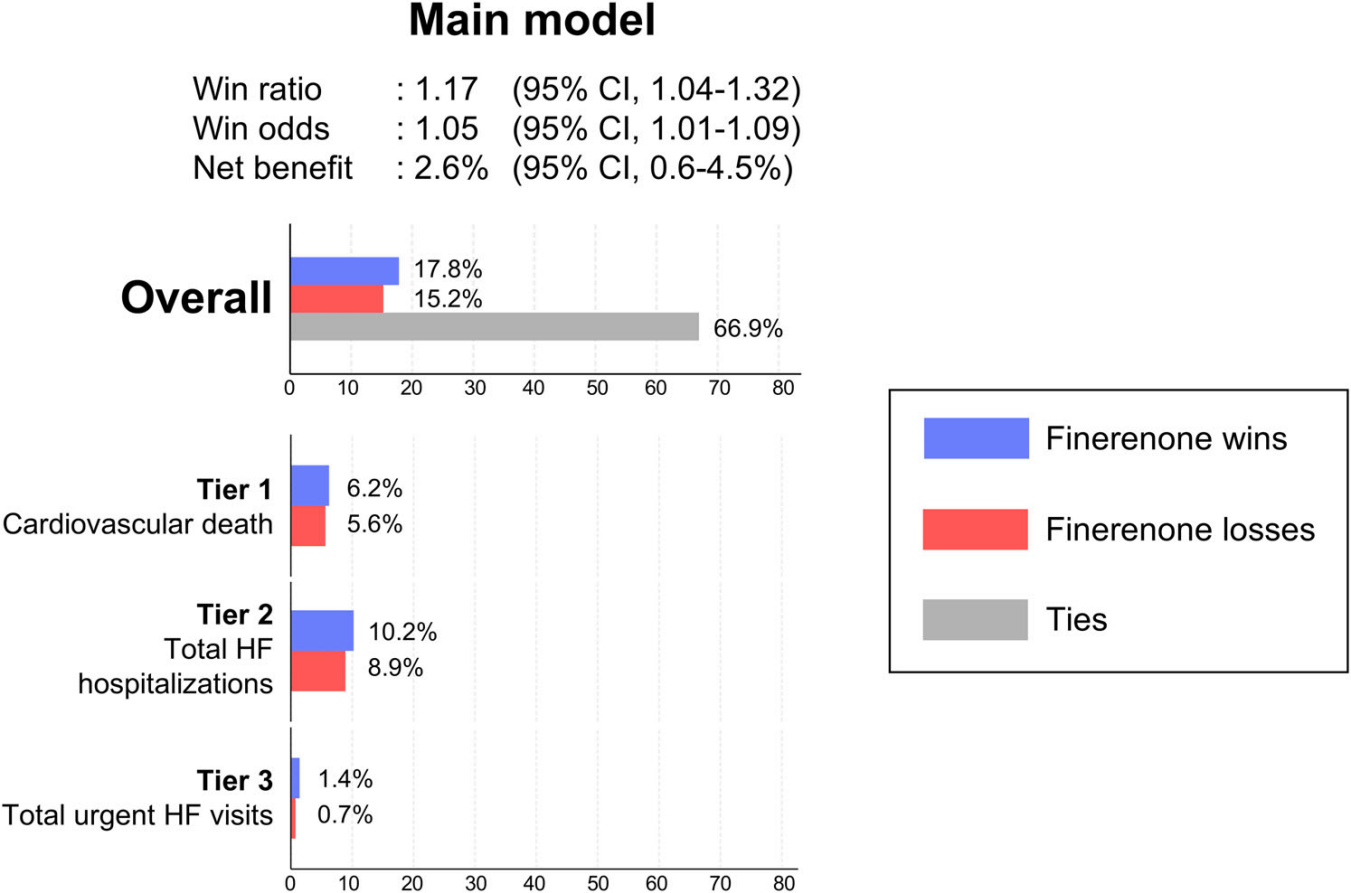
Effect of finerenone on the hierarchical composite endpoint at 24 months. The figure shows the effect of finerenone on the hierarchical composite endpoint evaluated by win statistics. Win statistics with 95% confidence interval (CI) are described. The horizontal bars at the top of the figure show the overall percentages of wins, losses, and ties in the finerenone group for the hierarchical composite endpoint. The groups of coloured bars below show the percentages of wins and losses for each tier included in the hierarchical composite endpoint. HF, heart failure.

In the main model evaluated at 24 months, the win ratio was 1.17 (95% CI 1.04–1.32) (*p* = 0.010), demonstrating more wins (17.8%) in the finerenone group than losses (15.2%) (*Figure* *1*). The win odds were 1.05 (95% CI 1.01–1.09), and the net benefit was 2.6% (95% CI 0.6–4.5%). The NNT with finerenone to prevent one primary outcome was 39 (online supplementary *Table* *S2*). Total HF hospitalizations (tier 2, wins 10.2% vs. losses 8.9%) contributed more to the overall results than cardiovascular death (tier 1; wins 6.2% vs. losses 5.6%). The percentage of pairs settled by total urgent HF visits (tier 3) was small at 2.1%, but the percentage of wins was two times larger than the percentage of losses (1.4% vs. 0.7%). The comparable analyses using the LWYY models, Cox proportional hazard models, and the mixed effect models are shown in *Table* *2*. There was stronger evidence of a beneficial effect of finerenone on total HF hospitalizations (RR 0.81 [95% CI 0.69–0.95]) and urgent HF visits (RR 0.64 [95% CI 0.45–0.90]) than there was for cardiovascular death (HR 0.91 [95% CI 0.74–1.11]).

**Figure 2:**
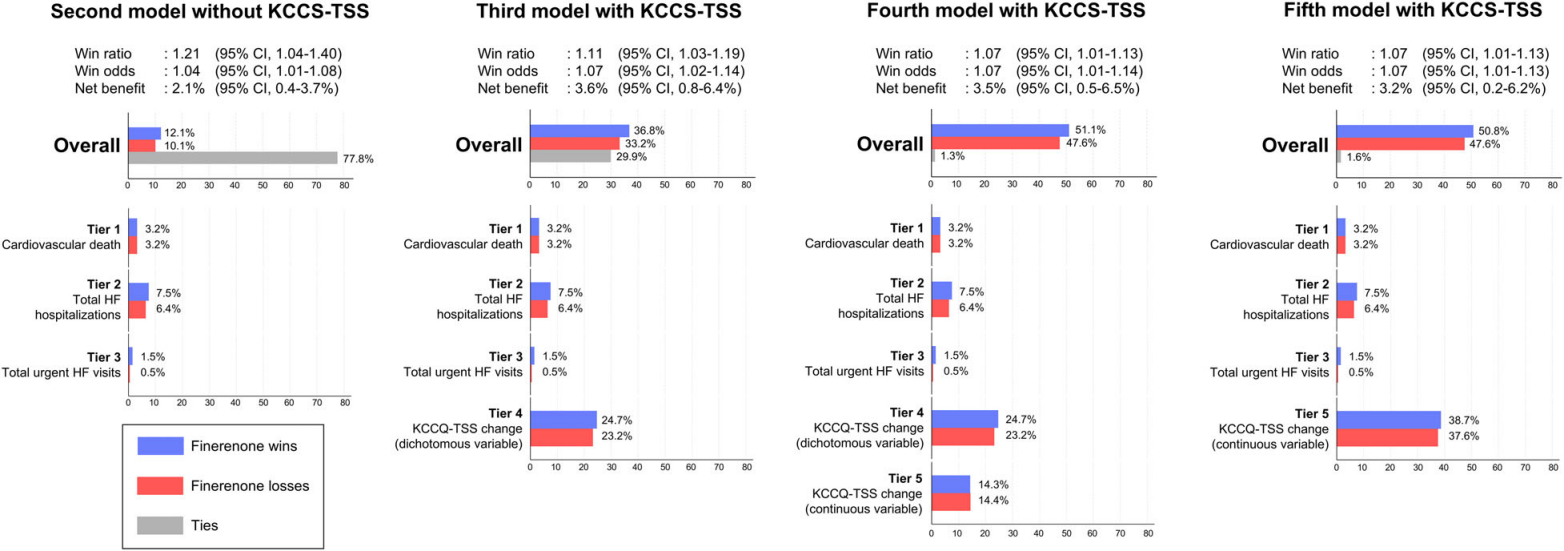
Effect of finerenone on the hierarchical composite endpoint with and without the Kansas City Cardiomyopathy Questionnaire total symptom score (KCCQ‐TSS) (12‐month data). The figure shows the effects of finerenone on the hierarchical composite endpoint in the models incorporating KCCQ (third, fourth, and fifth models) and the model without KCCQ‐TSS (second model). The KCCQ‐TSS component was included as a dichotomous variable (≥10‐point improvement, followed by ≥5‐point deterioration); as both a dichotomous variable (tier 4) and a continuous variable (tier 5, model 4); or just as a continuous variable (model 5). Win statistics with 95% confidence interval (CI) are described. The horizontal bars at the top of the figure show the overall percentages of wins, losses, and ties in the finerenone group for the hierarchical composite endpoint. The groups of coloured bars below show the percentages of wins and losses for each tier included in the hierarchical composite endpoint. HF, heart failure.

Compared to the main model, the percentage of ties was higher in the second model (77.8% in the second model vs. 66.9% in the main model) because of the shorter follow‐up duration, resulting in a smaller net benefit (2.6% vs. 2.1%) (*Figure* *2*). In the third model, 47.9% of pairs were settled by the KCCQ‐TSS tier, resulting in a 1.5% increase in net benefit, and the win odds increased from 1.04 to 1.07. Regardless of the model, adding KCCQ‐TSS to the hierarchical composite endpoint reduced the percentage of ties and increased the net benefit by 1.1% to 1.5% due to the higher percentage of wins (vs. losses) in the KCCQ‐TSS tier (third, fourth, and fifth models). In the sixth model evaluated with the full follow‐up, the net benefit increased from 2.6% to 3.0% with the same win ratio (i.e. 1.17) compared to the main model (online supplementary *Figure* *S1*).

### Win statistics over time

**Figure 3:**
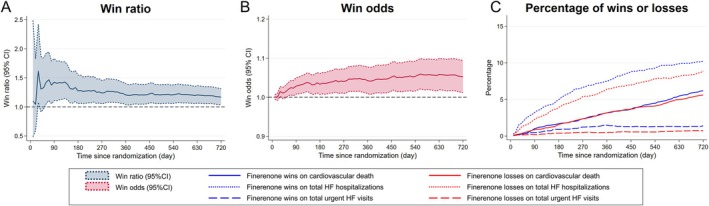
Win statistics over time. For the main model (i.e. hierarchy of tier 1, cardiovascular death; tier 2, total heart failure [HF] hospitalizations; tier 3, total urgent HF visits)¸ the following were plotted for every 10‐day fixed follow‐up period: win ratio with 95% confidence interval (CI) (*A*), the win odds with 95% CI (*B*), and the percentages of wins and losses (*C*).

*Figure* *3* shows the win statistics at every 10 days over follow‐up. The win ratio remained above 1.0 from 60 days (at 60 days, 1.43 [95% CI 1.06–1.93]) (*Figure* *3A*). The win odds increased gradually but steadily over time (*Figure* *3B*).

For cardiovascular death (tier 1), the percentages of wins and losses increased gradually and similarly up to 450 days, but after 450 days, the percentage of wins with finerenone increased over losses, widening the gap (*Figure* *3C*). Total HF hospitalizations (tier 2) contributed more to the overall results than cardiovascular death (tier 1) consistently after randomization. The number of pairs settled by the total urgent HF visits tier (tier 3) was lower than in the other tiers.

### Win statistics in subgroups

**Figure 4:**
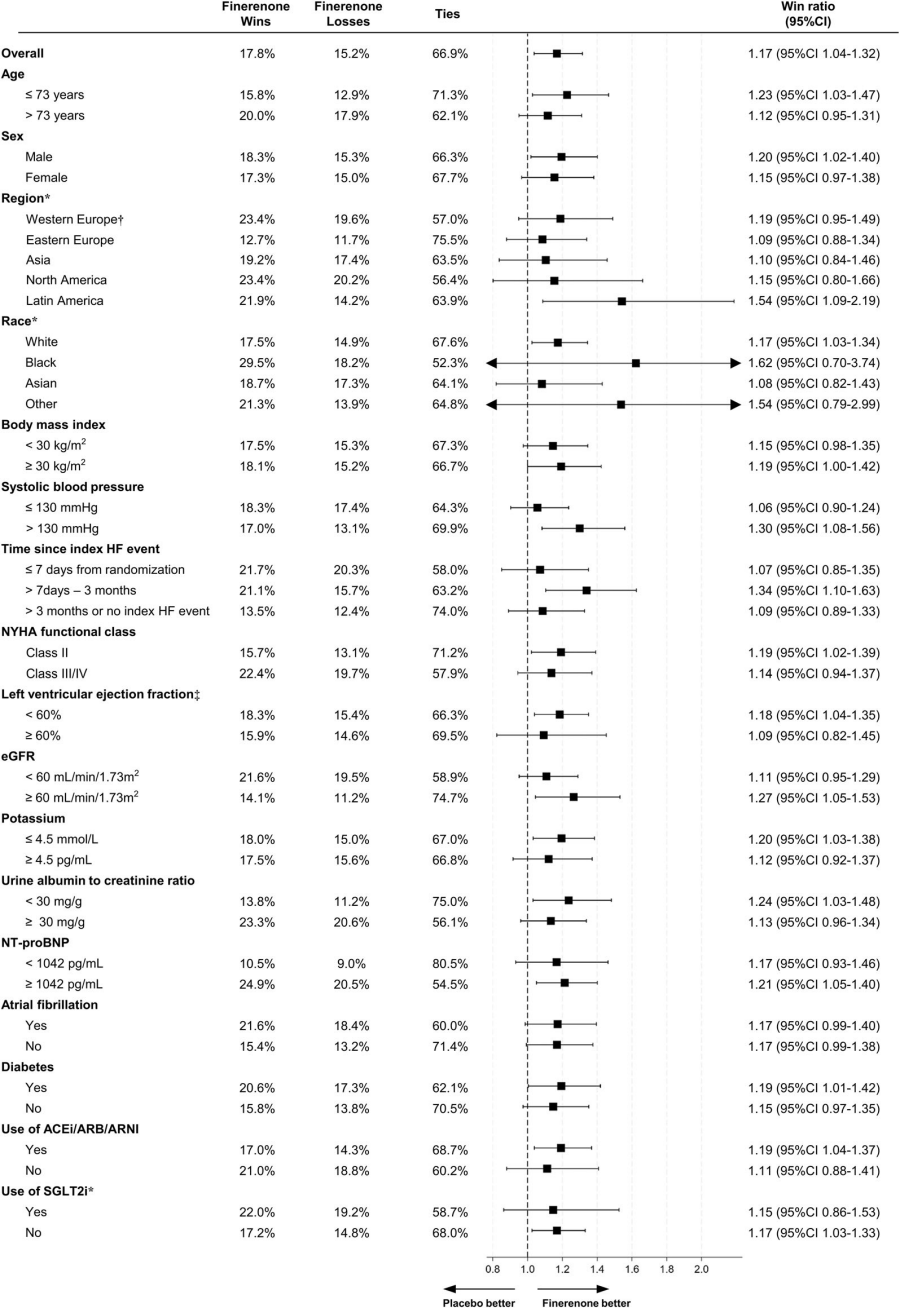
Effect of finerenone on the hierarchical composite endpoint according to selected subgroups. This figure shows win ratios and the percentages of wins, losses, and ties in the main model according to selected subgroups of patients. Win ratio was stratified by geographic region and baseline left ventricular ejection fraction (<60% or ≥60%). ACEi, angiotensin‐converting enzyme inhibitor; ARB, angiotensin receptor blocker; ARNI, angiotensin receptor–neprilysin inhibitor; CI, confidence interval; eGFR, estimated glomerular filtration rate; HF, heart failure; NT‐proBNP, N‐terminal pro‐B‐type natriuretic peptide; NYHA, New York Heart Association; SGLT2i, sodium–glucose cotransporter 2 inhibitor. *Stratified only by left ventricular ejection fraction (<60% or ≥ 60%). ^†^Including Oceania and others. ^‡^Stratified only by geographic region.

Win ratios and percentages of wins, losses, and ties in the finerenone group according to prespecified subgroups are shown in *Figure* *4*. The estimates of the win ratio were above 1.0 in all subgroups. Win odds for finerenone versus placebo according to subgroups are shown in online supplementary *Figure* *S2*.

## Discussion

In this prespecified analysis of FINEARTS‐HF, we showed that finerenone not only improved the primary outcome based on the original composite endpoint (i.e. total worsening HF events, including HF hospitalizations and urgent HF visits, as well as cardiovascular deaths) but also the corresponding hierarchical composite endpoint that takes into account of the clinical importance of these events (*Graphical Abstract*). The benefit of finerenone was observed early and increased over time. The initial benefit was primarily driven by a reduction in total HF hospitalizations, with the impact of cardiovascular death more pronounced later during the follow‐up period. Efficacy was observed in all subgroups. Finerenone also had a beneficial effect on an expanded hierarchical composite endpoint which incorporated the patient‐reported KCCQ‐TSS in addition to the other components.

As demonstrated in the present study, win statistics have several important advantages compared to conventional time‐to‐first event analyses. First, win statistics can handle both the number of events and time to events, while the Cox proportional hazard model, which is the most frequently used analytical approach in trials, does not account for recurrent events.^7^, ^8^, ^10^ Second, win statistics allow the creation of composite endpoints that take account of the clinical importance of the components of the composite, whereas both the Cox proportional hazard and semiparametric proportional rate method (e.g. LWYY) models cannot.^7^, ^8^, ^9^, ^10^ In the present analysis, we prioritized cardiovascular death as the most important event, followed by HF hospitalizations and urgent HF visits. Analysis of this hierarchical composite outcome confirmed the benefits of finerenone.

In this analysis of FINEARTS‐HF, the win ratio obtained from the ratio of wins to losses was 1.17 (95% CI 1.04–1.32) with 66.9% ties. Few other trials have evaluated the effect of drug treatment on a similar endpoint. One was DELIVER (Dapagliflozin Evaluation to Improve the Lives of Patients with Preserved Ejection Fraction Heart Failure), which showed that dapagliflozin compared with placebo in patients with HFmrEF/HFpEF gave a similar win ratio of 1.22 (95% CI 1.09–1.37) with 68.4% ties.^27^ Other win statistics allow for a comprehensive interpretation of treatment effects. Win odds, which is a modification of the win ratio that accounts for ties and corresponds to the odds that a random participant in the active treatment group has a better outcome than a random participant in the control group, gave a value of 1.05 (95% CI 1.01–1.09) and the net benefit, which corresponds to the absolute risk difference, was 2.6% (in DELIVER these values were 1.07 [95% CI 1.03–1.11] and 3.2%, respectively).^34^ In the presence of ties, it is preferable to interpret the effect based on win odds or net benefit because win ratio can overestimate the treatment effect. In addition, the NNT, calculated from the net benefit, may also help the clinical interpretation of win statistics, and for the primary analysis, it was 39 which is similar to the NNT in DELIVER (in a model with the same components) which was 32.^27^

The overall results in FINEARTS‐HF were driven more by the total and time‐to‐first hospitalization tier (difference in percentage of wins and losses, 1.3%) than by the cardiovascular death tier (difference, 0.6%), consistent with the RR or HR for the corresponding endpoints assessed by LWYY and the Cox proportional hazard models, respectively (RR 0.81 for total HF hospitalizations; HR 0.91 for cardiovascular death). Although fewer pairs were resolved by total and time‐to‐first urgent HF visits, they resulted in a 0.7% difference, which also aligns with the higher RR using the LWYY method (RR 0.64).

A further advantage of the win‐ratio approach is the ability to incorporate patient‐reported outcomes in a logical manner.^8^, ^10^ Incorporating the KCCQ‐TSS as an additional tier allowed comparison of pairs otherwise considered as ties after the three higher tiers (cardiovascular death, HF hospitalizations, and urgent HF visits) were analysed (thus this approach reduced the number of ties). As the optimal method for incorporating KCCQ‐TSS into the hierarchical composite endpoint has not been established, we analysed KCCQ‐TSS in different ways. Regardless of the specific method used, the addition of the KCCQ‐TSS as a fourth tier increased the net benefit obtained from finerenone by approximately 1.1–1.5% (and an increase was also seen with dapagliflozin in DELIVER).^6^, ^27^, ^43^ Similarly, the win odds, which corresponds to the treatment effect, increased from 1.04 to 1.07, while the win ratio decreased from 1.21 to 1.11 in the third model and fourth models and to 1.07 in the fifth model; this can be explained by the decrease in the percentage of ties due to incorporating KCCQ‐TSS. Used in this way, the expanded composite outcome demonstrated that finerenone provided an additional benefit by improving KCCQ‐TSS, even after accounting for the ‘harder’ outcomes in the earlier tiers (tiers 1–3). However, the increase in win odds or net benefit were lower in FINEARTS‐HF (win odds: from 1.04 to 1.07, net benefit: from 2.1% to 3.6%) compared to DELIVER (win odds: from 1.04 to 1.17, net benefit: from 2.2% to 7.9%) when adding KCCQ‐TSS to the hierarchical composite endpoint. This is in line with the difference in KCCQ‐TSS change between these trials; 1.6 points increase from baseline to 12 months in FINEARTS‐HF and 2.4 points increase from baseline to 8 months in DELIVER.^44^, ^45^

Win statistics are influenced by the follow‐up period, censoring distribution, and competing risks.^10^, ^31^, ^32^, ^33^, ^46^ The treatment effect on each component of the hierarchy can vary over time, leading to differences between short‐term and long‐term win statistics.^20^, ^33^ Therefore, observing changes in win statistics, along with the percentages of wins and losses at each tier level, over time can provide a deeper understanding of treatment effects. In the present study, the win ratio exceeded 1.0 early after randomization and reached a plateau of about 1.2 after approximately 12 months. The win odds increased gradually but steadily over time, indicating the benefit of finerenone over placebo accumulated over time. HF hospitalizations determined the overall win statistics early on, and the early benefit of finerenone was achieved through differences in HF hospitalizations and urgent HF visits. However, the impact of the cardiovascular death tier on overall win statistics becomes larger, over time, with a benefit of finerenone on cardiovascular death observed after 450 days.

Finally, the estimates of the win ratio for all subgroups exceeded 1.0 and these findings by subgroup were similar to the results for the primary outcome (total worsening HF events and cardiovascular deaths) evaluated using the LWYY method in the main analysis of FINEARTS‐HF.^6^

### Limitations

First, FINEARTS‐HF has variability in the censoring distribution due to its event‐driven study design. We addressed this by assessing win statistics after a fixed follow‐up period. However, this reduced the data available to analyse and results in an estimand that is ambiguously defined.^47^ It is not easy to speculate whether the censoring distribution and handling of it causes an overestimation or underestimation of the treatment effect. Second, although we included KCCQ‐TSS as the last tier in the hierarchical composite (as it was a predefined secondary endpoint), alternative outcomes could be considered for this tier. Third, there is no established standard approach as to how to include KCCQ‐TSS as a component in the hierarchical composite endpoint. However, we confirmed the validity and consistency of the results using different approaches.^48^ Fourth, we conducted a stratified analysis of the win statistics using the prespecified factors used in the analysis of the primary outcome in FINEARTS‐HF; however, due to a large number of subgroups, the number of patients in each stratum was small, leading to a reduction in pairs compared and consequent wide CIs of win statistics. Despite this, the effect of finerenone remained consistent across subgroups.

## Conclusions

Finerenone treatment significantly improved a composite hierarchical outcome, including cardiovascular death, total HF hospitalizations, and total urgent HF visits, with early onset of benefit. Incorporation of a patient‐reported outcome further augmented the benefit of finerenone.

### Funding

Dr. Kondo is supported by Great Britain Sasakawa Foundation, Yagi Foundation, and grant 20 K17112 from Grant‐in‐Aid for Scientific Research. Dr. Jhund and Dr. McMurray are supported by a British Heart Foundation Centre of Research Excellence Grant RE/18/6/34217 and the Vera Melrose Heart Failure Research Fund. Bayer was the sponsor and funder of the FINEARTS‐HF trial.

**Conflict of interest**: T.K. received lecture fees from Abbott Japan LLC, AstraZeneca K.K., Boehringer Ingelheim, Ono Pharmaceutical Co., Ltd., Kowa Company, Ltd, Kyowa Kirin Co., Ltd., and Novartis Pharma K.K. P.S.J. reports speakers' fees from AstraZeneca, Novartis, Alkem Metabolics, ProAdWise Communications, Sun Pharmaceuticals; advisory board fees from AstraZeneca, Boehringer Ingelheim, Novartis; research funding from AstraZeneca, Boehringer Ingelheim, Analog Devices Inc, Roche Diagnostics. P.S.J.'s employer the University of Glasgow has been remunerated for clinical trial work from AstraZeneca, Bayer AG, Novartis and Novo Nordisk. Director GCTP Ltd. A.D.H. has nothing to disclose. B.L.C. has received personal consulting fees from Alnylam, Bristol Myers Squibb, Cardior, Cardurion, Corvia, CVRx, Eli Lilly, Intellia, Rocket, and has served on a DSMB for Novo Nordisk. A.S.D. has received institutional research grants (to Brigham and Women's Hospital) from Abbott, Alnylam, AstraZeneca, Bayer, Novartis, and Pfizer as well as personal consulting fees from Abbott, Alnylam, AstraZeneca, Bayer, Biofourmis, Boston Scientific, Medpace, Medtronic, Merck, Novartis, Parexel, Porter Health, Regeneron, River2Renal, Roche, Veristat, Verily, Zydus. M.B. is a full‐time employee of Bayer AG. J.L.F. is a full‐time employee of Bayer plc, Research & Development, Pharmaceuticals, Reading, UK. P.S. is an employee of Bayer AG. P.V. is an employee of Bayer AG. F.A. is a full‐time employee of Bayer Pharmaceuticals. C.E.C. reports personal fees from AstraZeneca, Bayer, Boehringer Ingelheim, Daiichi‐Sankyo, MSD, Novartis, Pfizer, Sanofi, outside the submitted work. G.F. reports lecture fees from Bayer, Boehringer Ingelheim, Servier, Novartis, trial committee membership fees from Bayer, Boehringer Ingelheim, Servier, Impulse Dynamics, Vifor, Medtronic and consulting fees from Cardior, Novo Nordisk; research grants from the European Union. C.S.P.L. has received research support from NovoNordisk and Roche Diagnostics; has received consulting fees from Alleviant Medical, Allysta Pharma, AnaCardio AB, Applied Therapeutics, AstraZeneca, Bayer, Biopeutics, Boehringer Ingelheim, Boston Scientific, Bristol Myers Squibb, CardioRenal, CPC Clinical Research, Eli Lilly, Impulse Dynamics, Intellia Therapeutics, Ionis Pharmaceutical, Janssen Research & Development LLC, Medscape/WebMD Global LLC, Merck, Novartis, Novo Nordisk, Prosciento Inc, Quidel Corporation, Radcliffe Group Ltd., Recardio Inc, ReCor Medical, Roche Diagnostics, Sanofi, Siemens Healthcare Diagnostics and Us2.ai; and is a co‐founder & non‐executive director of Us2.ai. M.C.P. reports, outside of the submitted work, grants or contracts from Boehringer Ingelheim, Roche, SQ Innovations, AstraZeneca, Novartis, Novo Nordisk, Medtronic, Boston Scientific, Pharmacosmos; consulting fees from Akero, Applied Therapeutics, Amgen, AnaCardio, Biosensors, Boehringer Ingelheim, Novartis, AstraZeneca, Novo Nordisk, Abbvie, Bayer, Horizon Therapeutics, Foundry, Takeda, Cardiorentis, Pharmacosmos, Siemens, Eli Lilly, Vifor, New Amsterdam, Moderna, Teikoku, LIB Therapeutics, 3R Lifesciences; and is Director of Global Clinical Trials Partners. M.SE. has served on advisory boards, consultancy and honoraria for Novartis, Abbott, Merck, MSD, Vifor, AstraZeneca, Cardurion, Novo Nordisk, Bayer, Boehringer Ingelheim. M.Sc. reports other from Novo Nordisk, Novartis, AstraZeneca, Boehringer, outside the submitted work. S.V. reports grants and personal fees from Amarin, Amgen, AstraZeneca, Bayer, Boehringer Ingelheim, Eli Lilly, HLS Therapeutics, Novo Nordisk, Pfizer, PhaseBio, personal fees from Canadian Medical and Surgical Knowledge Translation Research Group, Janssen, Novartis, personal fees from S&L Solutions Event Management Inc, Sanofi, from null, outside the submitted work. A.A.V.'s employer received consultancy fees and/or research support from Adrenomed, Anacardio, AstraZeneca, Bayer AG, BMS, Boehringer Ingelheim, Corteria, EliLilly, Merck, Moderna, Novartis, Novo Nordisk, Roche diagnostics, SalubrisBio. D.v.L. receives support from Bayer to attend FINEARTS study meetings (virtual and face‐to‐face) and has served on advisory boards, consultancy and honoraria for Novartis, Daiichi‐Sankyo, Sanofi, Sanova, AstraZeneca, Novo Nordisk, Bayer, Boehringer Ingelheim, Vaxxinity and Recardio Inc, outside the submitted work. F.Z. reports personal fees from 89Bio, Abbott, Acceleron, Applied Therapeutics, Bayer, Betagenon, Boehringer, BMS, CVRx, Cambrian, Cardior, Cereno pharmaceutical, Cellprothera, CEVA, Inventiva, KBP, Merck, Novo Nordisk, Owkin, Otsuka, Roche Diagnostics, Northsea, USa2, having stock options at G3 Pharmaceuticals and equities at Cereno, Cardiorenal, Eshmoun Clinical research and being the founder of Cardiovascular Clinical Trialists. B.P. is a consultant for Bayer, AstraZeneca, Boehringer Ingelheim, Lexicon, Bristol Meyers Squibb, KBP Biosciences*, Sarfez Pharmaceuticals*, Pharmaceuticals*, SQinnovations*, G3 Pharmaceuticals, Sea Star Medical*, Vifor*, Prointel*, Brainstorm Medical* (*stock/stock options). US Patent 9 931 412‐site specific delivery of eplerenone to the myocardium, US Patent pending 63/045783 Histone modulating agents for the prevention and treatment of organ failure. M.V. has received research grant support, served on advisory boards, or had speaker engagements with American Regent, Amgen, AstraZeneca, Bayer AG, Baxter Healthcare, BMS, Boehringer Ingelheim, Chiesi, Cytokinetics, Fresenius Medical Care, Idorsia Pharmaceuticals, Lexicon Pharmaceuticals, Merck, Milestone Pharmaceuticals, Novartis, Novo Nordisk, Pharmacosmos, Relypsa, Roche Diagnostics, Sanofi, and Tricog Health, and participates on clinical trial committees for studies sponsored by AstraZeneca, Galmed, Novartis, Bayer AG, Occlutech, and Impulse Dynamics. S.D.S. has received research grants from Alexion, Alnylam, AstraZeneca, Bellerophon, Bayer, BMS, Boston Scientific, Cytokinetics, Edgewise, Eidos, Gossamer, GSK, Ionis, Lilly, MyoKardia, NIH/NHLBI, Novartis, Novo Nordisk, Respicardia, Sanofi Pasteur, Theracos, US2.AI and has consulted for Abbott, Action, Akros, Alexion, Alnylam, Amgen, Arena, AstraZeneca, Bayer, Boehringer Ingelheim, BMS, Cardior, Cardurion, Corvia, Cytokinetics, Daiichi‐Sankyo, GSK, Lilly, Merck, Myokardia, Novartis, Roche, Theracos, Quantum Genomics, Janssen, Cardiac Dimensions, Tenaya, Sanofi‐Pasteur, Dinaqor, Tremeau, CellProThera, Moderna, American Regent, Sarepta, Lexicon, Anacardio, Akros, Valo. J.J.V.M. reports payments through Glasgow University from work on clinical trials, consulting and grants from Amgen, AstraZeneca, Bayer, Cardurion, Cytokinetics, GSK and Novartis; personal consultancy fees from Alynylam Pharmaceuticals, Amgen, AnaCardio, AstraZeneca, Bayer, Berlin Cures, BMS, Cardurion, Cytokinetics, Ionis Pharmaceuticals, Novartis, Regeneron Pharmaceuticals, River 2 Renal Corp., British Heart Foundation, National Institute for Health—National Heart Lung and Blood Institute (NIH‐NHLBI), Boehringer Ingelheim, SQ Innovations, Catalyze Group; personal lecture fees from Abbott, Alkem Metabolics, AstraZeneca, Blue Ocean Scientific Solutions Ltd., Boehringer Ingelheim, Canadian Medical and Surgical Knowledge, Emcure Pharmaceuticals Ltd., Eris Lifesciences, European Academy of CME, Hikma Pharmaceuticals, Imagica Health, Intas Pharmaceuticals, J.B. Chemicals & Pharmaceuticals Ltd., Lupin Pharmaceuticals, Medscape/Heart.Org., ProAdWise Communications, Radcliffe Cardiology, Sun Pharmaceuticals, The Corpus, Translation Research Group, Translational Medicine Academy; DSMB: WIRB‐Copernicus Group Clinical Inc.; he is a director of Global Clinical Trial Partners Ltd.

## Supporting information


**Appendix S1.** Supporting Information.
