# Supplementary material for: The efficacy of finerenone on hierarchical composite endpoint analysed using win statistics in patients with heart failure and mildly reduced or preserved ejection fraction: A prespecified analysis of FINEARTS‐HF
Source: Eur J Heart Fail. 2025 Apr 29;27(8):1459–71. doi: 10.1002/ejhf.3669 (PMC12482849; doi:10.1002/ejhf.3669)
Supplement: Supplementary file 1 — Appendix S1. Supporting Information. [file EJHF-27-1459-s001.docx]

**SUPPLEMENTAL APPENDIX**

Online Supplement for manuscript entitled:

**The efficacy of finerenone on hierarchical composite endpoint analyzed using win statistics in patients with heart failure and mildly reduced or preserved ejection fraction: A prespecified analysis of FINEARTS-HF.**

**Supplementary Table 1. Summary of the hierarchical composite outcomes.**

| Outcome Model | Tier | Component of outcomes |
| --- | --- | --- |
| Main model | 1 | Cardiovascular death up at 24 months |
|  | 2 | Total HF hospitalizations at 24 months |
|  | 3 | Total urgent HF visits at 24 months |
| Second model | 1 | Cardiovascular death up at 12 months |
|  | 2 | Total HF hospitalizations at 12 months |
|  | 3 | Total urgent HF visits at 12 months |
| Third model* | 1 | Cardiovascular death up at 12 months |
|  | 2 | Total HF hospitalizations at 12 months |
|  | 3 | Total urgent HF visits at 12 months |
|  | 4 | KCCQ-TSS change from baseline to 12 months as a dichotomous variable (≥ 10 points deterioration, and next ≥ 5 points improvement) |
| Fourth model* | 1 | Cardiovascular death up at 12 months |
|  | 2 | Total HF hospitalizations at 12 months |
|  | 3 | Total urgent HF visits at 12 months |
|  | 4 | KCCQ-TSS change from baseline to 12 months as a dichotomous variable (≥ 10 points deterioration, and next ≥ 5 points improvement) |
|  | 5 | KCCQ-TSS change from baseline to 12 months as a continuous variable |
| Fifth model* | 1 | Cardiovascular death up at 12 months |
|  | 2 | Total HF hospitalizations at 12 months |
|  | 3 | Total urgent HF visits at 12 months |
|  | 4 | KCCQ-TSS change from baseline to 12 months as continuous variable |
| Sixth model | 1 | Cardiovascular death for the full follow-up |
|  | 2 | Total HF hospitalizations for the full follow-up |
|  | 3 | Total urgent HF visits for the full follow-up |

* Patients without KCCQ at baseline (n=15) were excluded from analysis.

HF, heart failure; KCCQ-TSS, Kansas City Cardiomyopathy Questionnaire - Total Symptom Score.

**Supplementary Table 2. Numbe needed to treat.**

|  | Number needed to treat |
| --- | --- |
| Main Model | 39 |
| Second model | 48 |
| Third model | 28 |
| Fourth model | 29 |
| Fifth model | 31 |
| Sixth model | 34 |

**Supplementary Figure 1. Effect of finerenone on the hierarchical composite endpoint for the full follow-up.**

**
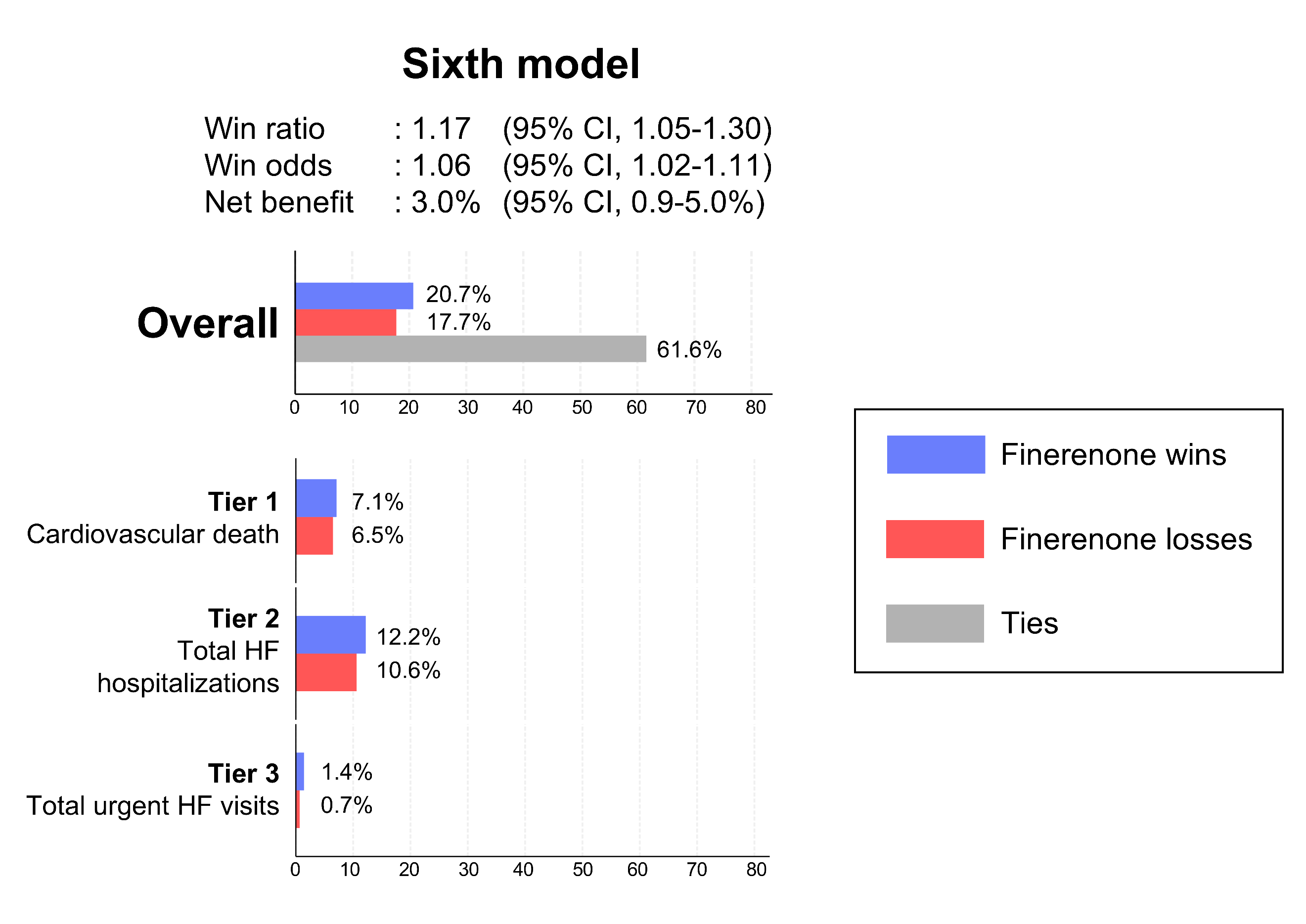
**

The figure shows the effect of finerenone on the hierarchical composite endpoint evaluated by win statistics. Win statistics with 95% CI are described. The horizontal bars at the top of the figure show the overall percentages of wins (blue), losses (red), and ties (gray) in the finerenone group for the hierarchical composite endpoint. The groups of colored bars below show the percentages of wins and losses for each tier included in the hierarchical composite endpoint.

CI, confidence interval; HF, heart failure.

**Supplementary Figure 2. Win odds according to selected subgroups.**


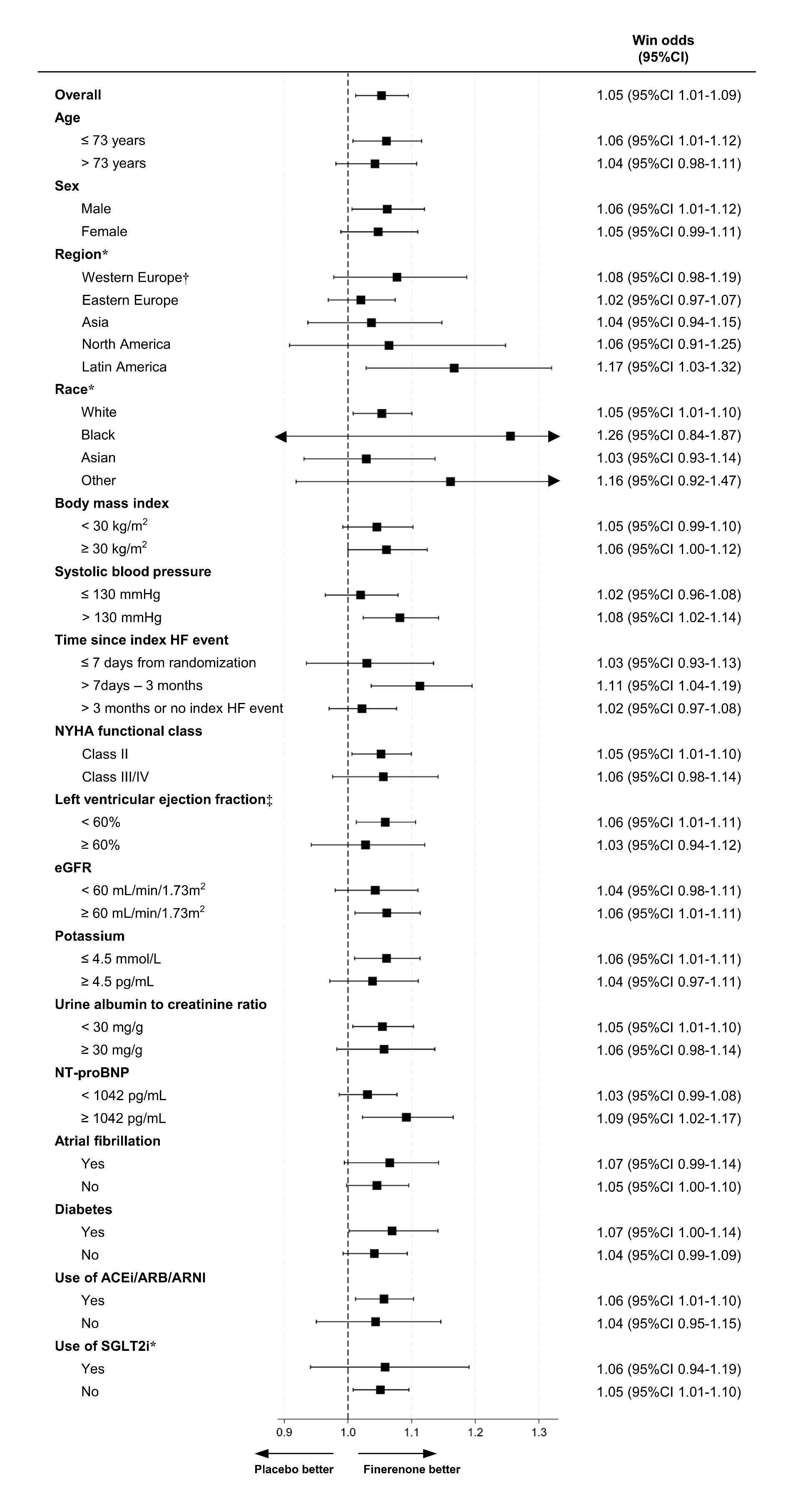


This figure shows win odds in the Main model according to selected subgroups of patients assigned to finerenone.

Win odds was stratified by geographic region and baseline LVEF (<60% or ≥60%).

* Stratified only by LVEF (<60% or ≥60%).

† Including Oceania and others.

‡ Stratified only by geographic region.

ACEi, angiotensin converting enzyme inhibitor; ARB, angiotensin receptor blocker; ARNI, angiotensin receptor neprilysin inhibitor; CI, confidence interval; eGFR, estimated glomerular filtration rate; HF, heart failure; NT-proBNP, N-terminal pro-B-type natriuretic peptide; NYHA, New York Heart Association; SGLT2i, sodium-glucose co-transporter-2 inhibitor.
